# Kankanet: An artificial neural network-based object detection smartphone application and mobile microscope as a point-of-care diagnostic aid for soil-transmitted helminthiases

**DOI:** 10.1371/journal.pntd.0007577

**Published:** 2019-08-05

**Authors:** Ariel Yang, Nahid Bakhtari, Liana Langdon-Embry, Emile Redwood, Simon Grandjean Lapierre, Patricia Rakotomanga, Armand Rafalimanantsoa, Juan De Dios Santos, Inès Vigan-Womas, Astrid M. Knoblauch, Luis A. Marcos

**Affiliations:** 1 School of Medicine, Stony Brook University, Stony Brook, New York, United States of America; 2 Global Health Institute, Stony Brook University, Stony Brook, New York, United States of America; 3 Immunopathology axis, Centre de Recherche du Centre Hospitalier de l'Université de Montréal, Montréal, Québec, Canada; 4 Mycobacteria Unit, Institut Pasteur de Madagascar, Antananarivo, Madagascar; 5 Helminthiasis Unit, Institut Pasteur de Madagascar, Antananarivo, Madagascar; 6 Department of Information Technology, Uppsala University, Uppsala, Sweden; 7 Immunology of Infectious Diseases Unit, Institut Pasteur de Madagascar, Antananarivo, Madagascar; 8 Department of Epidemiology and Public Health, Swiss Tropical and Public Health Institute, Basel, Switzerland; 9 Department of Medicine, Stony Brook University, New York, United States of America; National Institutes of Allergy and Infectious Diseases, NIH, UNITED STATES

## Abstract

**Background:**

Endemic areas for soil-transmitted helminthiases often lack the tools and trained personnel necessary for point-of-care diagnosis. This study pilots the use of smartphone microscopy and an artificial neural network-based (ANN) object detection application named Kankanet to address those two needs.

**Methodology/Principal findings:**

A smartphone was equipped with a USB Video Class (UVC) microscope attachment and Kankanet, which was trained to recognize eggs of *Ascaris lumbricoides*, *Trichuris trichiura*, and hookworm using a dataset of 2,078 images. It was evaluated for interpretive accuracy based on 185 new images. Fecal samples were processed using Kato-Katz (KK), spontaneous sedimentation technique in tube (SSTT), and Merthiolate-Iodine-Formaldehyde (MIF) techniques. UVC imaging and ANN interpretation of these slides was compared to parasitologist interpretation of standard microscopy.Relative to a gold standard defined as any positive result from parasitologist reading of KK, SSTT, and MIF preparations through standard microscopy, parasitologists reading UVC imaging of SSTT achieved a comparable sensitivity (82.9%) and specificity (97.1%) in *A*. *lumbricoides* to standard KK interpretation (97.0% sensitivity, 96.0% specificity). The UVC could not accurately image *T*. *trichiura* or hookworm. Though Kankanet interpretation was not quite as sensitive as parasitologist interpretation, it still achieved high sensitivity for *A*. *lumbricoides* and hookworm (69.6% and 71.4%, respectively). Kankanet showed high sensitivity for *T*. *trichiura* in microscope images (100.0%), but low in UVC images (50.0%).

**Conclusions/Significance:**

The UVC achieved comparable sensitivity to standard microscopy with only *A*. *lumbricoides*. With further improvement of image resolution and magnification, UVC shows promise as a point-of-care imaging tool. In addition to smartphone microscopy, ANN-based object detection can be developed as a diagnostic aid. Though trained with a limited dataset, Kankanet accurately interprets both standard microscope and low-quality UVC images. Kankanet may achieve sensitivity comparable to parasitologists with continued expansion of the image database and improvement of machine learning technology.

## Author summary

For rainforest-enshrouded rural villages of Madagascar, soil-transmitted helminthiases are more the rule than the exception. However, the microscopy equipment and lab technicians needed for diagnosis are a distance of several days’ hike away. We piloted a solution for these communities by leveraging resources the villages already had: a traveling team of local health care workers, and their personal Android smartphones. We demonstrated that an inexpensive, commercially available microscope attachment for smartphones could rival the sensitivity and specificity of a regular microscope using standard field fecal sample processing techniques. We also developed an artificial neural network-based object detection Android application, called Kankanet, based on open-source programming libraries. Kankanet was used to detect eggs of the three most common soil-transmitted helminths: *Ascaris lumbricoides*, *Trichuris trichiura*, and hookworm. We found Kankanet to be moderately sensitive and highly specific for both standard microscope images and low-quality smartphone microscope images. This proof-of-concept study demonstrates the diagnostic capabilities of artificial neural network-based object detection systems. Since the programming frameworks used were all open-source and user-friendly even for computer science laymen, artificial neural network-based object detection shows strong potential for development of low-cost, high-impact diagnostic aids essential to health care and field research in resource-limited communities.

## Introduction

Soil-transmitted helminths (STH) such as *Ascaris lumbricoides*, hookworm, and *Trichuris trichiura* affect more than a billion people worldwide [1–3]. However, due to lack of access to fecal processing materials, diagnostic equipment, and trained personnel for diagnosis, the mainstay of STH control remains mass administration of antihelminthic drugs [4]. To diagnose STH in residents of rural areas, the present standard is the Kato-Katz technique (estimated sensitivity of 0.970 for *A*. *lumbricoides*, 0.650 for hookworm, and 0.910 for *T*. *trichiura*; estimated specificity of 0.960 for *A*. *lumbricoides*, 0.940 for hookworm, and 0.940 for *T*. *trichiura*) [5]. However, this method is time-sensitive due to rapid degeneration of hookworm eggs [5]. Other methods, including fecal flotation through FLOTAC and mini-FLOTAC still have higher sensitivity (0.440) than direct fecal examination (0.360), but require centrifugation equipment, which is expensive and difficult to transport [6]. Multiplex quantitative PCR analysis for these three species is a high sensitivity and specificity technique (0.870–1.00 and 0.830–1.00, respectively), but can only be performed with expensive laboratory equipment [7,8]. Spontaneous sedimentation technique in tube (SSTT) analysis has been found in preliminary studies to be not inferior to Kato-Katz in *A*. *lumbricoides*, *T*. *trichiura*, and hookworm [9,10]. Since it requires no special equipment and few materials, it has the potential to be a cost-effective stool sample processing method in the field.

Mass drug administration campaigns are the prevailing strategy employed to control high rates of STH. Such campaigns, however, are focused on treating children and do not necessarily address the high infection prevalence rates of STH in adults, which in turn may contribute to the high reinfection rates [11,12]. Technology that facilitates point-of-care diagnosis could enable mass drug administration programs to screen adults for treatment, monitor program efficacy, aid research, and map STH prevalence. In areas close to STH elimination, such a tool could facilitate a test-and-treat model for STH control.

One avenue for point-of-care diagnostic equipment is smartphone microscopy. Numerous papers have already demonstrated the viability of using smartphones [13–15] and smartphone-compatible microscopy attachments (USB Video Class, or UVC) [16] as cheap point-of-care diagnostic tools. Studies have tried direct imaging, as with classical parasitological diagnosis [17], fluorescent labeling [14], and digital image processing algorithms to aid diagnosis [18].

To address the need for trained parasitologists to make the STH diagnosis, this study investigated artificial neural network-based technology (ANN). ANN, a framework from machine learning, a subfield of artificial intelligence, has seen a rapid explosion in range of applications, from object detection to speech recognition to translation. Rather than traditional software, which relies on a set of human-written rules for image classification, a method explored in other studies [19], ANN image processing stacks thousands of images together and uses backpropagation, a recursive algorithm to create its own rules to classify images. A previous study has applied ANN-based systems to diagnostic microscopy of STH with moderate sensitivity, using a device of comparable price to a smartphone to image samples and applying a commercially available artificial intelligence algorithm (Web Microscope) to classify the samples. However, such a device requires internet connection to function and was only validated on 13 samples [20,21]. Another study has created and patented an ANN-based system to identify *T*. *trichiura* based on a small dataset of sample images (n<100) [22]. However, there is no precedent in current literature for extensive (n>1,000) ANN-based object detection system training for multiple STH species, nor use in smartphones, nor offline use (disconnected from the internet), nor field testing in specimens. This study developed such a system, named Kankanet from the English word *network* and the Malagasy word for intestinal worms, *kankana*. This study also uses a smartphone-compatible mobile microscope, or UVC, with a simple X-Y slide stage.

As a proof-of-concept pilot study for ANN-assisted microscopy, this project aimed to address two key obstacles to point-of-care diagnosis of STH in rural Madagascar: (1) the lack of portable and inexpensive microscopy, and (2) the limited capacity and expertise to read microscope images. This project evaluated the efficacy for diagnosis of three species of STH of (1) a UVC and (2) Kankanet, an object-detection ANN-based system deployed through smartphone application.

## Methods

### Ethical considerations

This study was a part of a larger study on the "Assessment of Integrated Management for Intestinal Parasites control: study of the impact of routine mass treatment of Helminthiasis and identification of risk areas of transmission in two villages in the district of Ifanadiana, Madagascar". This study has received institutional review board approval from the Stony Brook University (ID: 874952–13) and the national ethics review board of Madagascar: Comité d’Éthique de la Recherche Biomédicale Auprès du Ministère de la Santé Publique de Madagascar (41-MSANP/CERBM, June 8, 2017). As a prospective study, data collection was planned before any diagnostic test was performed. In accordance with cultural norms, consent was first required from the local leaders before engaging in any activities within their purview. All participants received oral information about the study in Malagasy; written informed consent was obtained from adult participants or parents/legal guardians for the children.

Since this study was meant to evaluate diagnostic methods and did not produce definitive results, no diagnostic results from this study were reported to the patients. All inhabitants of the two study villages were given their annual dose of 400 mg albendazole one year before this study, and received another 400 mg albendazole dose within a month of the conclusion of the study by the national mass drug administration effort.

### Data storage

A unique identifier was assigned to each participant to allow grouping of analysis data for each patient. All data was stored on an encrypted server, to which only investigators had access.

### Study area, study population, and subject recruitment

The two villages under study, Mangevo and Ambinanindranofotaka (geographic coordinates: 21°27'S, 47°25'E and 21°28'S, 47°24'E), are rural villages situated on the edge of Ranomafana National Park, about 275 km south of Antananarivo, the capital of Madagascar. Over 95% of households in Ambinanindranofotaka (total population, n = 327) and Mangevo (total population, n = 238) engage in subsistence farming and animal husbandry. The villages, accessible only by 14 hours’ worth of footpaths, are tucked between mountain ridges covered with secondary-growth rainforest. The study was conducted between 8 Jun 2018 and 18 Jun 2018.

All residents of each village were given a brief oral presentation about the public health importance, symptoms and prevention of STH; subjects above age 16, the Madagascar cut-off age for adulthood, who gave voluntary consent to participate in the study were given containers and gloves to collect their own fecal samples. Parents gave consent for their assenting children and collected their fecal samples. One fecal sample from each participant was submitted between the hours of sunrise and sunset. Samples were processed for analysis within 20 minutes of production by participant. Cognitively impaired subjects were excluded.

### Fecal sample processing

Each fecal sample produced three slides for microscopic analysis: (1) one slide was prepared according to Kato-Katz (KK) technique from fresh stool; (2) one slide was prepared according to spontaneous sedimentation technique in tube (SSTT) from 10% formalin-preserved stool; (3) one slide was prepared according to Merthiolate-Iodine-Formaldehyde (MIF) technique from 10% formalin-preserved stool.

As a reference test, a modified gold standard was defined as any positive result (at least one egg positively identified in a sample) from standard microscopy by trained parasitologists using (1) KK, (2) SSTT, and (3) MIF techniques. Intensity of infection (measured by eggs/gram) of *A*. *lumbricoides*, *T*. *trichiura*, and hookworm were obtained by standard microscopy reading of KK slides by multiplying the egg count per slide reading by the standard coefficient of 24. SSTT technique followed standard protocol [23]. This measure was defined to increase the sensitivity of the reference test.

**Fig 1:**
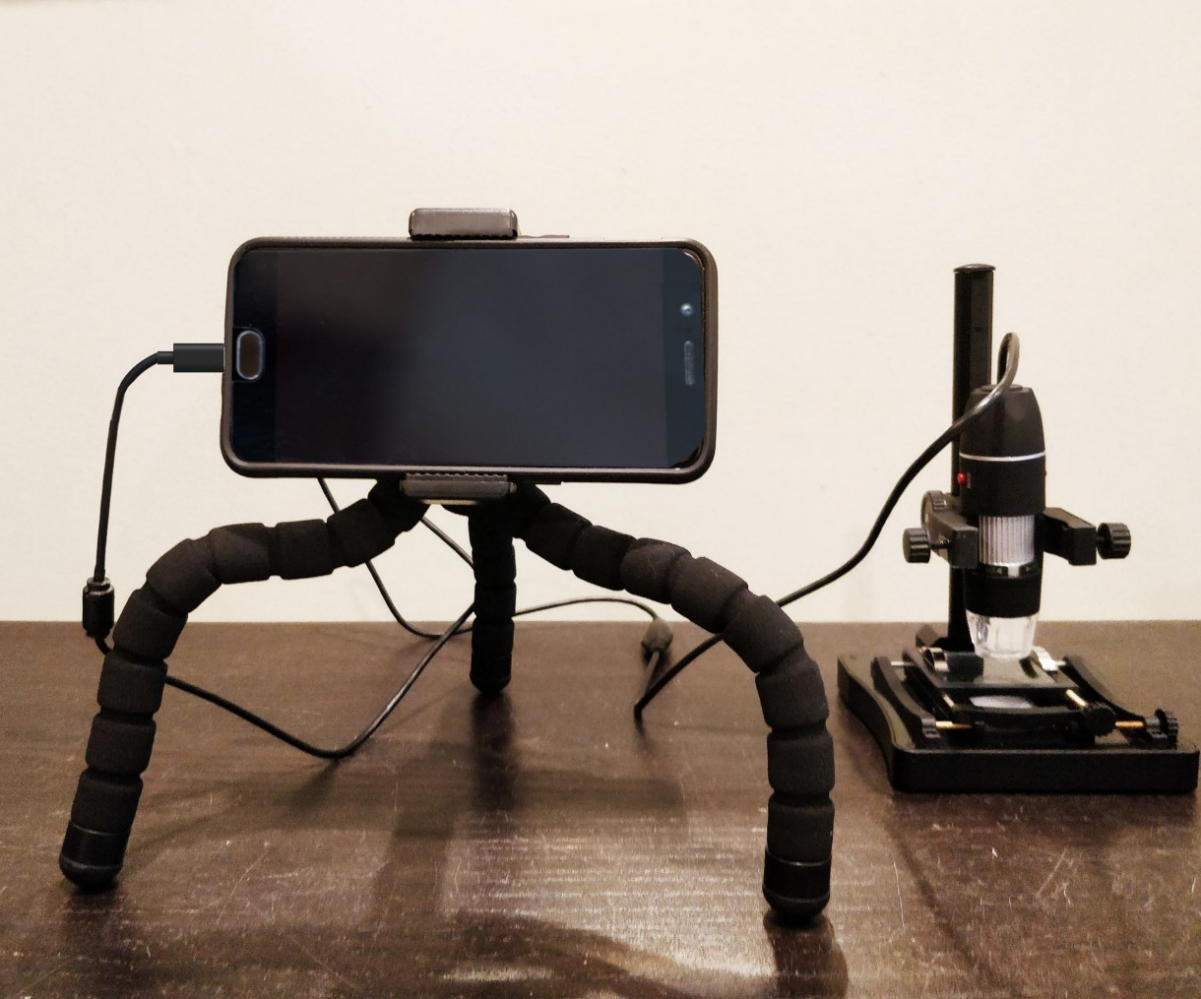
An Android smartphone mounted on a tripod connected to the UVC used in this study, which is mounted in a microscope stand with X-Y stage.

A standard Android smartphone was attached to a UVC (Magnification Endoscope, Jiusion Tech; Digital Microscope Stand, iTez) for microscopic analysis of KK and SSTT slides in the field (Fig 1). Clinical information or results from any other analyses of the fecal samples was not made available to slide readers during their analysis.

### Object detection ANN-based system training

TensorFlow is an open-source machine learning framework developed by Google Brain. Using the TensorFlow repository, this study developed Kankanet, an ANN-based object detection system built upon a Single Shot Detection meta-architecture and a MobileNet feature extractor, a convolutional neural network developed for mobile vision applications [24,25]. Based on a dataset of 2,078 images of STH eggs, Kankanet was trained to recognize three STH species: *A*. *lumbricoides*, *T*. *trichiura*, and hookworm [26]. 597 egg pictures were taken by a standard microscope and 1,481 were taken by UVC. The efficacy of Kankanet diagnosis was evaluated with a separate dataset of 186 images with a comparable distribution of species and imaging modalities. The detailed breakdown of the composition of these image sets is shown in Table 1, which shows percentage distributions by species and imaging modality to show concordance in image distribution between training set and evaluation set.

**Table 1:** Distribution of images of *A. lumbricoides*, *T. trichiura*, and hookworm in each image dataset, taken with each modality. The training set and evaluation set are composed of similar distributions of each species, with similar breakdowns of imaging modality.

	Training set % (n)	Evaluation set % (n)
**AL total**	**90.1% (1872/2078)**	**86.6% (161/186)**
** AL microscope**	19.4% (404**/**2078)	23.1% (43/186)
** AL UVC**	70.6% (1468**/**2078)	63.4% (118/186)
**TT total**	**4.8% (100/2078)**	**9.1% (17/186)**
** TT microscope**	4.2% (87**/**2078)	5.9% (11/186)
** TT UVC**	0.6% (13**/**2078)	3.2% (6/186)
**H total**	**5.1% (106/2078)**	**4.3% (8/186)**
** H microscope**	5.1% (106**/**2078)	4.3% (8/186)
** H UVC**	0.0% (0**/**2078)	0.0% (0/186)
**Total images**	**100.0% (2078)**	**100.0% (186)**
** Total microscope**	28.7% (597**/**2078)	33.3% (62/186)
** Total UVC**	71.3% (1481**/**2078)	66.7% (124/186)

**Fig 2:**
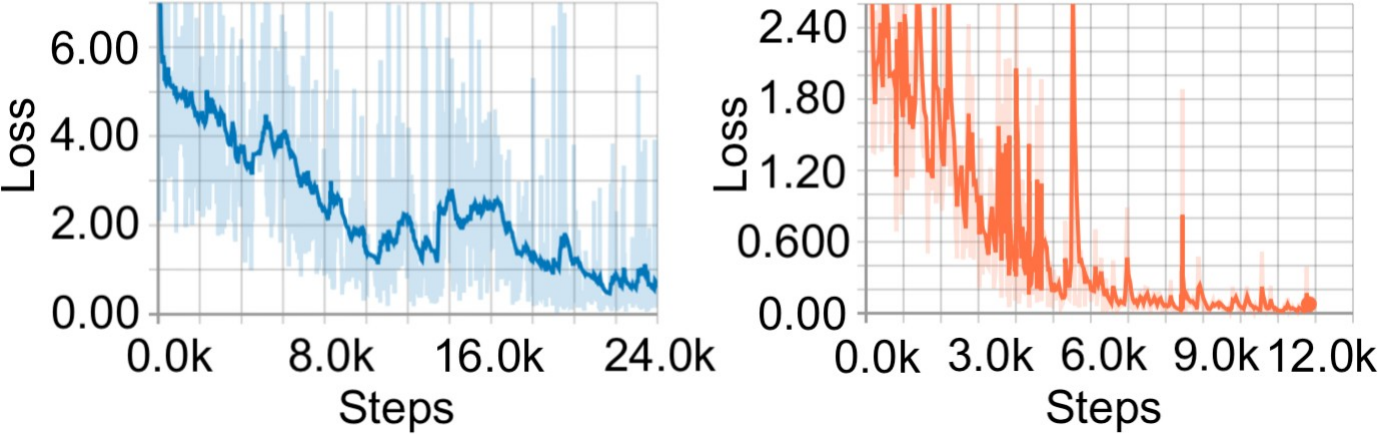
Loss as it evolved over the course of training Model 1 (blue) and Model 2 (orange).

The following hyperparameters were used: initial learning rate = 0.004; decay steps = 800720; decay factor = 0.95, according to the default configuration used to train open-source models released online. To improve the robustness of the model, the dataset was augmented using the default methods of random cropping and horizontal flipping. The loss rate was monitored until it averaged less than 0.01, as shown in Fig 2, after which the model was frozen in a format suitable for use in a mobile application. Based on this protocol, two models were trained:

1. Model 1, trained with microscope images only (n = 597).
2. Model 2, trained with all microscope and UVC images (n = 2,078).

**Fig 3:**
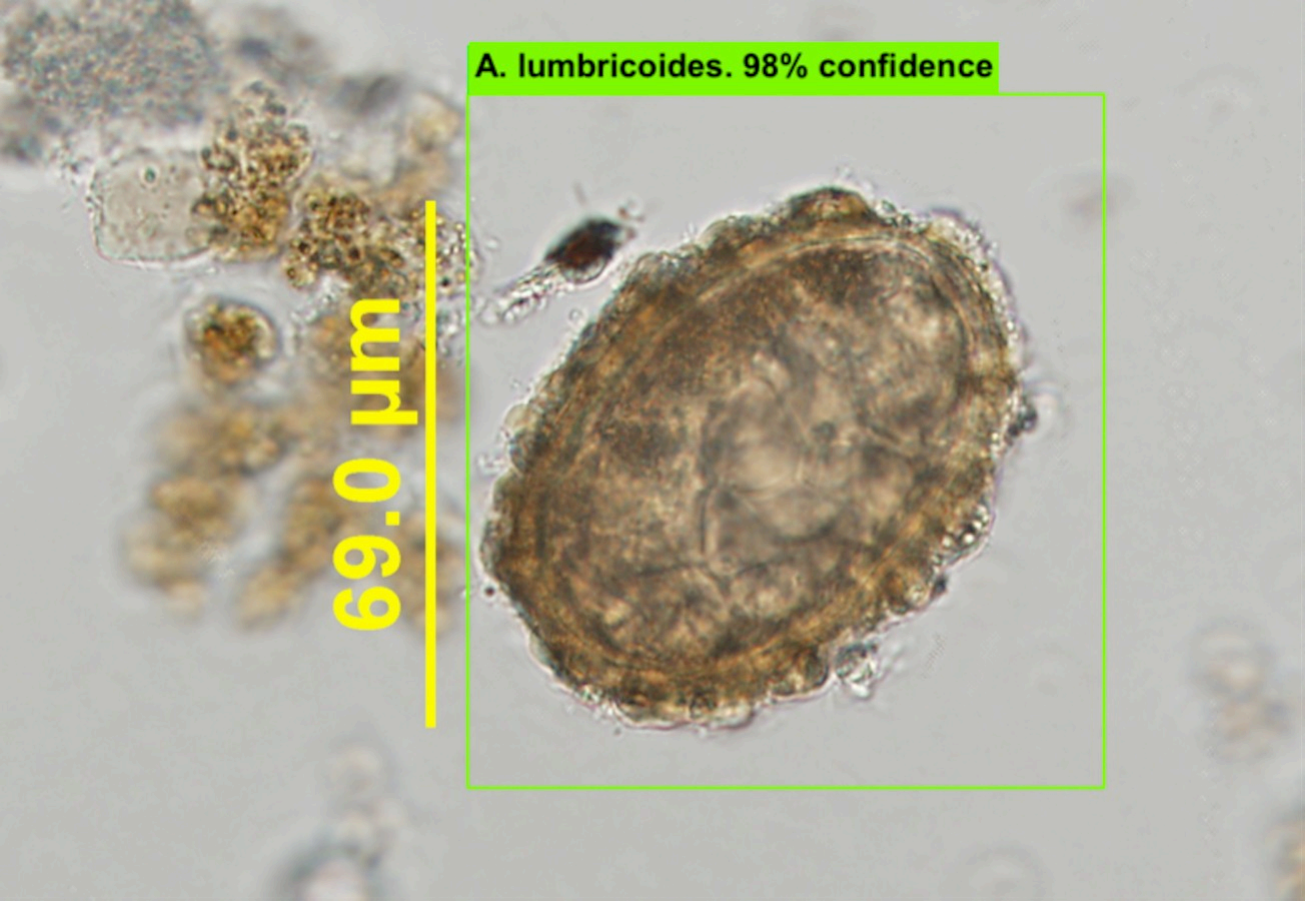
Detected *A*. *lumbricoides* egg using Model 1 on a standard microscope picture.

**Fig 4:**
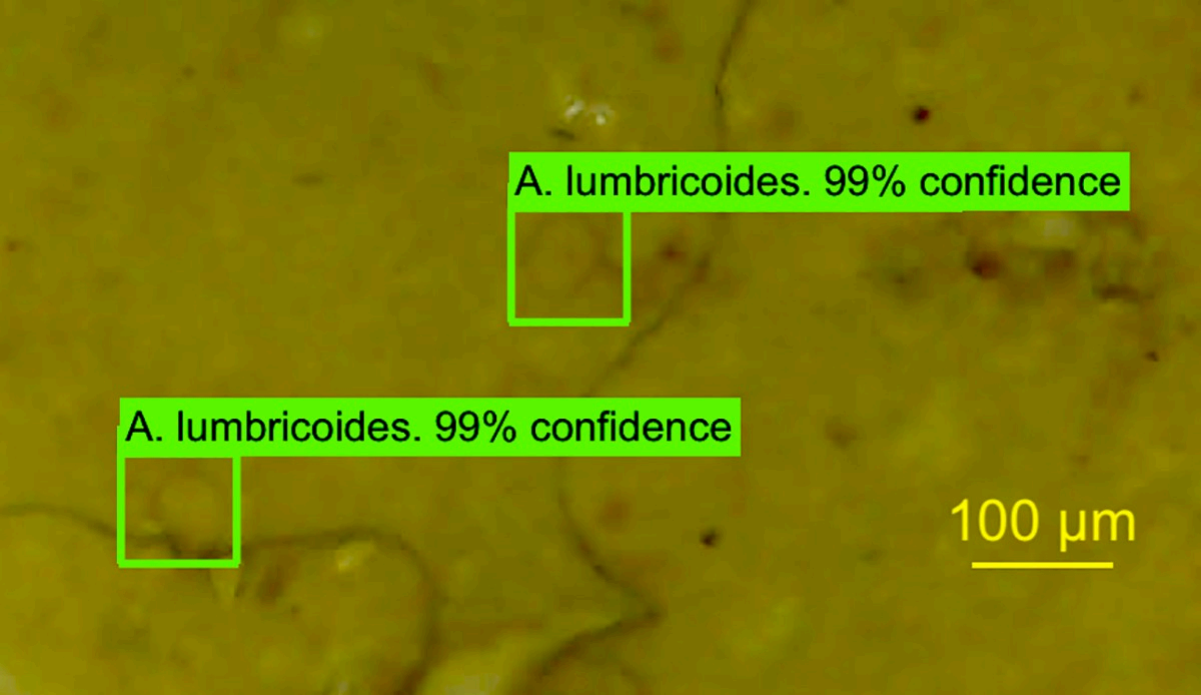
Detected *A*. *lumbricoides* eggs using Model 2 on a UVC picture.

It took Model 1 around 81 and Model 2 around 12 epochs, or iterations through the entire training dataset, to reach the loss rate of less than 0.01. These models were then validated by being tested from randomly selected images from the evaluation image set (n = 185), images that were not included in the training set. Once trained, these models analyze images in real time, project a bounding-box over each detected object, and display the name of the object detected, along with a confidence rating (Fig 3 and Fig 4).

The true readings of each image in the training and test image sets were determined by a trained parasitologist. The Kankanet models then were used to read test set images, and correctly identified eggs were considered true positives, incorrect objects identified as eggs were considered false positives, undetected eggs were considered false negatives, and images without eggs or detected objects were considered true negatives. Evaluation of model sensitivity and specificity was performed with the following test image sets:

1. UVC images (n = 124)
2. microscope images (n = 62)
3. both UVC and microscope images (n = 186)

### Android application development

The open-source TensorFlow library contains a demo Android application that includes an object-detection module. Following the protocol for migrating this TensorFlow model to Android [27], the original object detection model on the app was swapped out for the Kankanet model. As per the original app, the threshold for reporting detected objects was set at 0.60 confidence.

### Data analysis

Intended sample size was calculated based on June 2016 prevalence rates in Ifanadiana, Madagascar (n = 574): *A*. *lumbricoides* 71.3% (95% CI 67.7–75.1); *T*. *trichiura* 74.7% (95% CI 71.1–78.2); hookworm 33.1% (95% CI 29.2–36.9) [28]. Following the calculations for a binary diagnostic test for the species with the lowest prevalence, hookworm, with a predicted sensitivity of the test of 90% and a 10% margin of error, the required sample size to have adequate power was determined to be 115. For *A*. *lumbricoides* and *T*. *trichiura*, which have higher prevalence rates, a sample size of 115 gave sufficient power to support a sensitivity of 70% with a margin of error of 10%. This study used a sample size of 113 fecal samples.

Readings from the UVC on KK and SSTT slides were compared against the modified gold standard, which is defined as any positive result from a standard microscopy reading of KK, SSTT, and MIF techniques by a parasitologist. In SPSS, sensitivity and specificity of the UVC reading were calculated for each species with KK, SSTT, and combined analysis. Separate analyses were calculated for different intensities of infection as classified according to WHO guidelines [4]. Cohen’s Kappa coefficient (K) was calculated for each type of fecal processing method to determine comparability to the modified gold standard reading.

Results from Kankanet interpretation were compared to visual interpretation of the same images by a trained parasitologist. The two models were evaluated for sensitivity, specificity, positive predictive value, and negative predictive value using SPSS. There were no samples that had missing results from any of the tests run.

## Results

### Gold standard readings and intensity of infection measurements

The number of positive samples identified by standard microscopy through the Kato-Katz, MIF, and SSTT preparation methods are shown in Table 2, as well as the composite reading used as the modified gold standard in this study of the three tests. The number of samples of *A*. *lumbricoides* and *T*. *trichiura* at each intensity level is reported in Table 3. There were no participants heavily infected with *T*. *trichiura*. Since it was not possible for the KK slides to be transported to the laboratory in time for quantification of hookworm eggs, we were unable to detect the intensity of infection of these cases.

**Table 2:** Positive samples identified by standard microscopy of KK, MIF, and SSTT, and the modified gold standard, considered as any positive from the three standard assays.

Positive results with assay, n				
	KK	MIF	SSTT	Modified gold standard
***A*. *lumbricoides***	78	73	65	79
***T*. *trichiura***	99	39	26	101
**Hookworm**	0*	4	18	22

KK, Kato-Katz technique; MIF, merthiolate-iodine-formaldehyde technique; SSTT, spontaneous sedimentation technique in tube

*Kato-Katz readings could not be obtained for hookworm due to disintegration of the eggs in Kato-Katz slides during transport to lab facility

**Table 3:** The breakdown of positive samples of *A*. *lumbricoides and T*. *trichiura* by WHO intensity of infection categories, as measured by standard microscopy of Kato-Katz preparations.

	*A*. *lumbricoides*		*T*. *trichiura*	
	n	%	n	%
**Low**^**a**^	33	29.2	76	67.3
**Moderate**^**b**^	42	37.2	23	20.4
**Heavy**^**c**^	3	2.7	0	0.0

^a^1-4999 epg *A*. *lumbricoides*, 1–999 epg *T*. *trichiura*

^b^5000-49 999 epg *A*. *lumbricoides*, 1000–9999 epg *T*. *trichiura*

^c^≥50 000 epg *A*. *lumbricoides*, ≥10 000 epg *T*. *trichiura*

### UVC analysis

**Fig 5:**
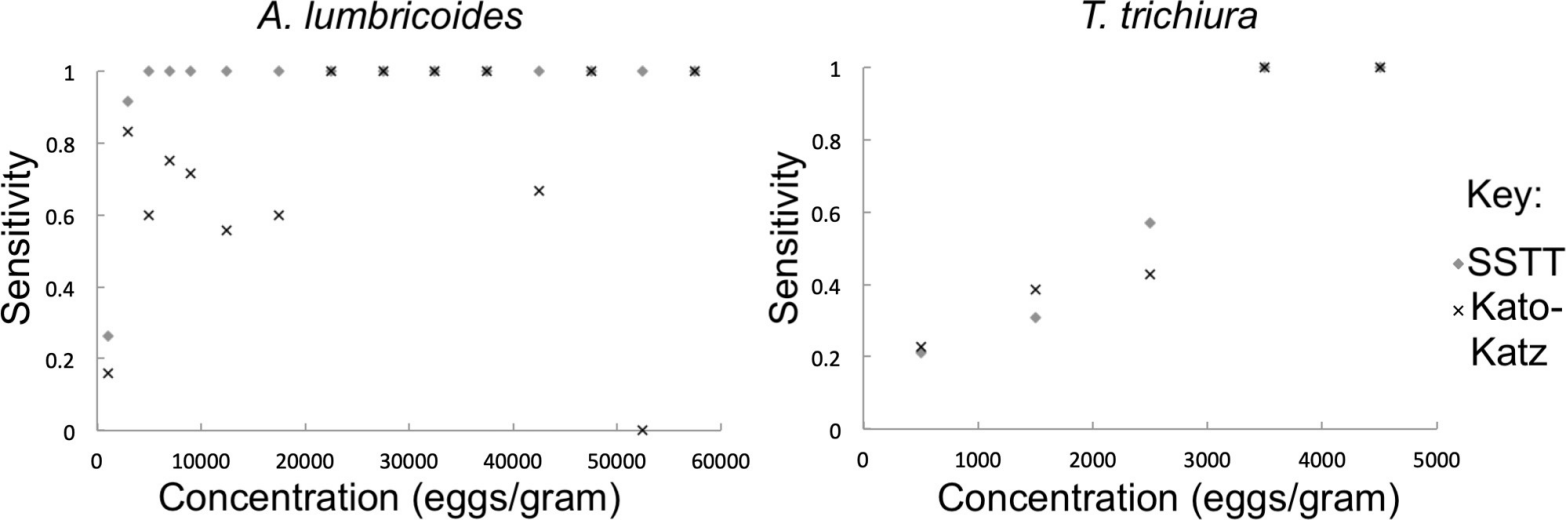
Sensitivity of UVC readings of SSTT and KK preparations for *A*. *lumbricoides* (left) *and T*. *trichiura* (right) increase with increasing intensity of infection.

The UVC performed best at imaging *A*. *lumbricoides* (Tables 4 and 5), demonstrating higher sensitivity in SSTT preparations (0.829, 95% CI .744-.914) than in KK (0.579, 95% CI .468-.690), and high specificity in both SSTT and KK (0.971, 95% CI .915–1.03; 0.971, 95% CI .915–1.03). These sensitivity numbers increased with increasing infection intensity (Fig 5). UVC imaging of SSTT slide preparations of samples with AL showed a substantial level of concordance with the modified gold standard reading, which was obtained through standard microscopy (K = 0.728), and UVC imaging of KK slide preparations demonstrated moderate concordance with the modified gold standard (K = 0.439). For *T*. *trichiura*, the UVC demonstrated low overall sensitivity through SSTT and KK (0.224, 95% CI .141-.307; 0.235, 95% CI .151-.319, respectively), but high specificity (0.917, 95% CI .761–1.07; 1, 95% CI 1.00–1.00). As infection intensity of *T*. *trichiura* increased, however, sensitivity increased (Fig 5). According to WHO categories for infection intensity, sensitivity for low-intensity infections was 0.164, which increased to 0.435 in moderate-intensity infections. There was little agreement with the modified gold standard (K = 0.038 for SSTT, K = 0.063 for KK). The UVC also demonstrated low sensitivity to hookworm eggs in both SSTT (0.318, 95% CI .123-.513) and KK (0.381, 95% CI .173-.589) preparations.

**Table 4:** The sensitivity, specificity, and Cohen's Kappa values of UVC readings by trained parasitologist against the modified gold standard using SSTT.

		UVC with SSTT					
		*A*. *lumbricoides*		*T*. *trichiura*		Hookworm	
		Negative	Positive	Negative	Positive	Negative	Positive
**Modified gold standard**	Negative	33	1	11	1	85	3
	Positive	13	63	76	22	15	7
**Kappa**		0.728		0.038		0.355	
**Sensitivity**		0.829, 95% CI .744-.914		0.224, 95% CI .141-.307		0.318, 95% CI .123-.513	
**Specificity**		0.971, 95% CI .915–1.03		0.917, 95% CI .761–1.07		0.966, 95% CI .928–1.00	

**Table 5:** The sensitivity, specificity, and Cohen's Kappa values of UVC readings by trained parasitologist against the modified gold standard using KK.

		UVC with Kato-Katz					
		*A*. *lumbricoides*		*T*. *trichiura*		Hookworm	
		Negative	Positive	Negative	Positive	Negative	Positive
**Modified gold standard**	Negative	33	1	12	0	64	25
	Positive	32	44	75	23	13	8
**Kappa**		0.439		0.063		0.403	
**Sensitivity**		0.579, 95% CI .468-.690		0.235, 95% CI .151-.319		0.381, 95% CI .173-.589	
**Specificity**		0.971, 95% CI .915–1.03		1, 95% CI 1.00–1.00		0.719, 95% CI .637-.821	

### Kankanet model analysis

Model 1, which was trained and evaluated on microscope images only, demonstrated high sensitivity (1.00; 95% CI 1.00–1.00) and specificity (0.910; 95% CI 0.831–0.989) for *T*. *trichiura*, low sensitivity (0.571; 95% CI 0.423–0.719) and specificity (0.500; 95% CI 0.275–0.725) for *A*. *lumbricoides*, and low sensitivity (0.00; 95% CI 0.00–0.00) and specificity (0.800; 95% CI 0.693–0.907) for hookworm. Table 6 shows the full breakdown of sensitivity, specificity, positive predictive value, and negative predictive value of the different analyses performed by Model 1 and Model 2. Though Model 1 was also evaluated for its performance on UVC pictures of STH, it failed to recognize any, and thus the results are not tabulated. Model 2 was trained on images taken both with microscopes and with UVC, and was tested with both types of images. It outperformed Model 1 in every parameter, with high sensitivity and specificity for microscope images all across the board and for UVC images of *A*. *lumbricoides* and hookworm. It performed poorly on UVC images of *T*. *trichiura* (sensitivity 0.093, 95% CI -0.138–0.304; specificity 0.969, 95% CI 0.934–1.00), but had moderate PPV and NPV values (0.667 and 0.800, respectively).

**Table 6:** Sensitivity, specificity, positive predictive value, and negative predictive value of Model 1 and Model 2, by types of images used.

		Model 1				Model 2			
Evaluation set	Species	Se (%)	Sp (%)	PPV (%)	NPV (%)	Se (%)	Sp (%)	PPV (%)	NPV (%)
**Microscope images**	**AL**	57.1	50.0	44.4	62.5	85.7	87.5	85.7	87.5
	**TT**	100.0	91.0	80.0	100.0	100.0	100.0	100.0	100.0
	**H**	0.0	80.0	0.0	61.5	66.7	100.0	100.0	80.0
**UVC images**	**AL**	0.0	N/A	N/A	N/A	68.5	40.0	92.5	10.5
	**TT**	0.0	N/A	N/A	N/A	8.3	96.9	50.0	73.8
	**H**	0.0	N/A	N/A	N/A	100.0	100.0	100.0	100.0
**All images**	**AL**	0.0	N/A	N/A	N/A	69.6	61.1	92.0	23.9
	**TT**	0.0	N/A	N/A	N/A	15.4	97.8	66.7	80.0
	**H**	0.0	N/A	N/A	N/A	71.4	100.0	100.0	96.2

AL, *A*. *lumbricoides*; H, hookworm; N/A, not applicable; NPV, negative predictive value; PPV, positive predictive value; Se, sensitivity; Sp, specificity; TT, *T*. *trichiura*.

Model 1 failed to identify any eggs in UVC images.

## Discussion

This study found that UVC imaging of SSTT slides, though of low quality, still could be read by trained parasitologists with a high sensitivity (0.829, 95% CI .744-.914) and specificity (0.971, 95% CI .915–1.03) in *A*. *lumbricoides*, which is comparable to literature estimates of KK sensitivity at 0.970 and specificity of 0.960 [5]. The UVC showed lower sensitivity for KK preparations (0.579, 95% CI .468-.690). This UVC does not have sufficient image quality to be used with *T*. *trichiura* or hookworm diagnosis, which have thinner and more translucent membranes.

Despite UVC imaging having high sensitivity for *A. lumbricoides*, the 14% difference in sensitivity needs improvement, with a goal of reaching similar sensitivity to standard microscopy, before it can be feasibly used in large-scale STH control efforts. UVC’s specificity of 0.971 (95% CI 0.915–1.03) surpasses that of standard microscopy KK’s 0.960 specificity. Though currently shown to have insufficient sensitivity or specificity for use with *T*. *trichiura* or hookworm diagnosis, these are limitations believed to be related to the particular microscope peripheral used in this study. This UVC achieved maximum magnification of approximately 215X at 600 px/mm; its resolution was 640x480 pixels. The magnification level with this peripheral is sufficient, as other studies have shown success with *T*. *trichiura* with magnification levels as low as 60X [29]. However, for the purposes of STH imaging, improvement of resolution and light source in this UVC may be necessary. Another study successfully imaged *T*. *trichiura* and hookworm at a resolution of 2595x1944 pixels, which is substantially higher than the 640x480 with this peripheral [20]. This UVC’s light source comes from the same direction as the camera, rather than shining through the sample as in most microscopy, which may have reduced image quality and imaging ability.

Development of a proprietary microscope is another solution, which many other studies have employed: a mobile phone microscope developed by Coulibaly et al. has demonstrated similarly high sensitivity for *Schistosoma mansoni* (0.917; 95% CI 0.598–0.996), *Schistosoma haematobium* (0.811; 95% CI 0.712–0.883) and *Plasmodium falciparum* (0.802, 1.00) [30,31]; other studies that employ ball lenses or low-cost foldable chassis show slightly lower sensitivity/specificity values [29,32]. Independent development of a smartphone microscope could substantially improve the sensitivity and specificity of these devices to an acceptable level for healthcare use, that is, not inferior to standard microscopy, while simultaneously decreasing the cost per microscope. However, the advantage of using a commercially available microscope is ease of access for rapid, large-scale implementation and feasibility for low-income rural areas with a heavy burden of STH.

In the context of these villages in rural Madagascar, where STH prevalence can be as high as 93.0% for *A*. *lumbricoides*, 55.0% for *T*. *trichiura*, and 27.0% for hookworm as measured in 1998 [33], yet only school-aged children receive for mass drug administration, a rule-in test with high specificity, which this UVC achieves, can be useful to reliably identify adults who would also require antihelminthics. Another context in which this tool may be especially useful is areas close to elimination of STH, to reduce the amounts of antihelminthics needed for STH control [34].

Though Kankanet interpretation of UVC and microscope images yielded lower sensitivity than trained parasitologist readings of these images, Kankanet Model 2 still achieved high sensitivity for *A*. *lumbricoides* (0.696; 95% CI 0.625–0.767) and hookworm (0.714; 95% CI 0.401–1.027) on both microscope and UVC images. Model 2 showed high sensitivity for *T*. *trichiura* in microscope images (1.00; 95% CI 1.00–1.00), but low in UVC images (0.083; 95% CI -0.138–0.304). Model 1 achieved lower sensitivity and specificity for all species, and could not accurately interpret UVC images.

Model 2’s overall sensitivity for *A*. *lumbricoides*, *T*. *trichiura*, and hookworm (0.696, 0.154, and 0.714, respectively) may not seem very high at first. However, these are sensitivity results given for recognizing individual eggs. As an indication for treatment with antihelminthics would only require one egg per fecal sample slide to be positively identified, the real likelihood of this ANN-based object detection model giving an accurate reading is much higher than the per-egg sensitivity cited here. For example, even in an infection of *A*. *lumbricoides* at the middle of the range considered low-intensity (2500 eggs per gram), a slide would contain 104 eggs, making the sensitivity of detection of infection in the slide nearly 1.00.

The difference in sensitivity and specificity between the models can be explained by the differences in image sets used for training. Model 2 was trained with an image set of over twice the size of Model 1’s image set; Model 2’s image set also contained images from both UVC and standard microscopy modalities. It was a robust model, accurately detecting STH in images with multiple examples of multiple species, despite being trained on an image set containing mostly *A*. *lumbricoides*. It demonstrated a very low rate of false positives, considering the amount of debris apropos to fecal samples. The Kankanet models can be improved by developing a larger image dataset, exploring other object detection meta-architectures, and optimizing file size and computational requirements. A greater number and more even distribution of images of parasite species would improve object detection model sensitivity.

Standard laboratory processing and diagnosis of STH is extremely time-consuming and expensive and hence, not often practical for rural low-income communities. As smartphone penetrance will only increase in the coming years, medical technology should leverage smartphones as portable computational equipment, as use and distribution of such software requires no additional cost. Because it is able to be attached to smartphones and requires no external power source than the smartphone itself, UVC is a suitable microscopy option for point-of-care diagnosis. In addition, the smartphone application used in this study did not require internet access, unlike those of previous studies [20].

UVC and Kankanet are cost-effective, with only the initial cost of $69.82 for the microscope and stage setup, as well as the negligible cost of fecal analysis reagents. In the case of SSTT, only microscope slides and Lugol’s iodine would be needed for fecal processing. These initial costs are readily defrayed by the thousands of analyses performed with just one unit, the work-hours gained by timely treatment of STH and prevention of STH re-infection, and the reduction of unnecessary drug administration and concomitant drug resistance. A detailed cost analysis comparing the cost of standard microscopy and the Kankanet system for 2-sample Kato-Katz testing of 10 villages in rural Madagascar (estimated 3000 people total) is shown in Table 7. Whereas standard microscopy ends up costing around 1.33 USD per person tested, the Kankanet system costs around 0.56 USD per person tested.

**Table 7:** Estimated cost (in USD) for a team of 4 health care workers to survey 10 villages, or 3000 2-slide Kato Katz tests, using standard and Kankanet methods.

	Standard	Kankanet
**Kato-Katz training**	**3.78**	**3.78**
**Slide interpretation training**	**7.56**	**1.26**
Transportation to villages*	**800**	**400**
Time in villages*	**400**	**1000**
**Time spent analyzing outside of village**	**1000**	**0**
**Laboratory usage**^%^	**110**	**0**
**Equipment**^#^	**1671.96**	**279.28**
**Total cost for 10 villages**	**3993.3**	**1684.32**
**Price per 2-slide Kato-Katz test**	**1.3311**	**0.56144**

*Using the research center standard health care worker daily salary of 18,000 Ariary (approx. 5 USD), and hourly rate of 2,250 Ariary (approx. 0.63 USD)

^%^Using the research center station fee rate of 2,000 Ariary per person per day (approx. .55 USD)

^#^Using the standard Amscope 40X-2000X Binocular Biological Microscope with Mechanical Stage, 417.99 USD, and price for Kankanet setup (Jiusion Microscope and Aven X-Y microscope stand), 69.82 USD

ANN-based object detection systems such as the one introduced here can be useful for screening STH-endemic communities in the context of research, mass drug administrations and STH mapping programs. In addition, Kankanet, rather than replacing human diagnosis, could be a useful diagnostic training aid for healthcare workers and field researchers. With sustained use of such a tool, these workers may more quickly learn how to identify such eggs themselves.

Limitations of this study include that the UVC used was of insufficient image quality to produce accurate imaging of *T*. *trichiura* and hookworm. The Kankanet models employed used a dataset limited to two imaging modalities: standard microscopy and UVC, and with images of only three species of STH; in addition, images for this dataset were only taken of samples prepared under KK conditions, so the efficacy of this system can only be assessed for those conditions.

We conclude that parasitologist interpretation of UVC imaging of SSTT slides can be a field test comparable to standard microscopy of KK for *A*. *lumbricoides*. Second, we conclude that ANN interpretation is a feasible avenue for development of a point-of-care diagnostic aid. With 85.7% sensitivity and 87.5% specificity for *A*. *lumbricoides*, 100.0% sensitivity and 100.0% specificity for *T*. *trichiura*, and 66.7% sensitivity, 100.0% specificity for hookworm, Kankanet Model 2 has demonstrated stellar results in interpreting UVC images, even though it was trained with a limited proof-of-concept dataset. We hope that continued expansion of the Kankanet image database, improved imaging technology, and improvement of machine learning technology will soon enable Kankanet to achieve rates comparable to those of parasitologists.

## Supporting information

S1 Checklist Checklist for studies of diagnostic tests. (DOCX)

S1 Chart Flow chart for studies of diagnostic tests. (PDF)
