# Supplementary material for: Kankanet: An artificial neural network-based object detection smartphone application and mobile microscope as a point-of-care diagnostic aid for soil-transmitted helminthiases
Source: PLoS Negl Trop Dis. 2019 Aug 5;13(8):e0007577. doi: 10.1371/journal.pntd.0007577 (PMC6695198; doi:10.1371/journal.pntd.0007577)
Supplement: S1 Chart — (PDF) [file pntd.0007577.s002.pdf]

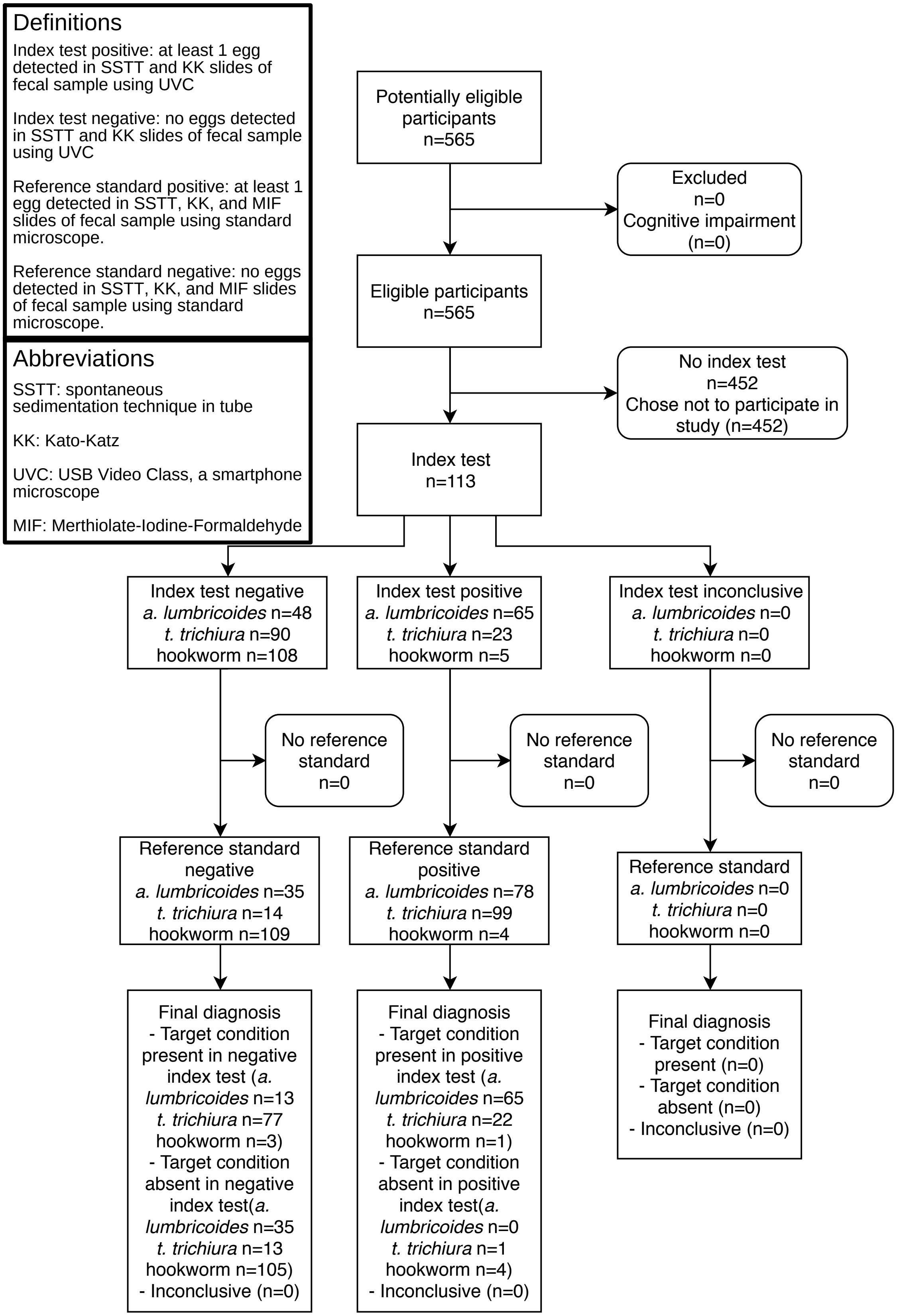
Definitions
Index test positive: at least 1 egg detected in SSTT and KK slides of fecal sample using UVC
Index test negative: no eggs detected in SSTT and KK slides of fecal sample using UVC
Reference standard positive: at least 1 egg detected in SSTT, KK, and MIF slides of fecal sample using standard microscope.
Reference standard negative: no eggs detected in SSTT, KK, and MIF slides of fecal sample using standard microscope.
Abbreviations
SSTT: spontaneous sedimentation technique in tube
KK: Kato-Katz
UVC: USB Video Class, a smartphone microscope
MIF: Merthiolate-Iodine-Formaldehyde
Potentially eligible participants n=565
Excluded n=0 Cognitive impairment (n=0)
Eligible participants n=565
No index test n=452 Chose not to participate in study (n=452)
Index test n=113
Index test negative *a. lumbricoides* n=48 *t. trichiura* n=90 hookworm n=108
Index test positive *a. lumbricoides* n=65 *t. trichiura* n=23 hookworm n=5
Index test inconclusive *a. lumbricoides* n=0 *t. trichiura* n=0 hookworm n=0
No reference standard n=0
No reference standard n=0
No reference standard n=0
Reference standard negative *a. lumbricoides* n=35 *t. trichiura* n=14 hookworm n=109
Reference standard positive *a. lumbricoides* n=78 *t. trichiura* n=99 hookworm n=4
Reference standard *a. lumbricoides* n=0 *t. trichiura* n=0 hookworm n=0
Final diagnosis - Target condition present in negative index test (*a. lumbricoides* n=13 *t. trichiura* n=77 hookworm n=3) - Target condition absent in negative index test(*a. lumbricoides* n=35 *t. trichiura* n=13 hookworm n=105) - Inconclusive (n=0)
Final diagnosis - Target condition present in positive index test (*a. lumbricoides* n=65 *t. trichiura* n=22 hookworm n=1) - Target condition absent in positive index test(*a. lumbricoides* n=0 *t. trichiura* n=1 hookworm n=4) - Inconclusive (n=0)
Final diagnosis - Target condition present (n=0) - Target condition absent (n=0) - Inconclusive (n=0)
